# The impact of apparent temperature on the emergency visits for traumatic fractures in Hangzhou, China

**DOI:** 10.1186/s12889-024-19119-z

**Published:** 2024-06-24

**Authors:** Feng Li, Xuejiao Liu, Yanlin Niu, Jinghong Gao, Maoqiang Li, Yipin Zhao, Cheng Ji, Guobiao Pan, Mingxing Zhao, Boliang Wu, Xiaoxiang Tang, Gang Wu, Jun Tian, Jianwei Chen, Shiyu Yan, Jianlu Tan, Yunqing Li, Wentao Zhao, Lingyun Li, Yinmiao Qiu, Wangxiang Yao, Liulong Zhu

**Affiliations:** 1 Department of Orthopedics, The Third People’s Hospital of Xiaoshan Hangzhou, Hangzhou, 311251 China; 2 Department of Medical Record Management and Statistics, Beijing Jishuitan Hospital, Capital Medical Univisity, Beijing, 100035 China; 3 Beijing Center for Disease Prevention and Control, Institute for Nutrition and Food Hygiene, Beijing, 100035 China; 4 Institute for Hospital Management of Henan Province, The First Affiliated Hospital of Zhengzhou University, Zhengzhou, Henan Province 450000 China; 5 Department of Orthopedics, Hangzhou First People’s Hospital, Zhejiang University School of Medicine, Hangzhou, 310006 China; 6 Department of Orthopedics, Third People’s Hospital of Hangzhou, Hangzhou, 310009 China; 7 Department of orthopedics, The First People’s Hospital of Yuhang District, Hangzhou, Hangzhou, 311199 China; 8 Department of Orthopedics, The Second People’s Hospital of Jiande, Hangzhou, 311600 China; 9 Department of Orthopedics, The Second People’s Hospital of Fuyang District, Hangzhou, Hangzhou, 311400 China; 10 Department of orthopedics, Affiliated Hospital of Jining Medical University, Jining, 272007 China; 11 Nanjing University of Chinese Medicine, Nangjing, 210023 China; 12 Department of orthopedics, Beijing Shunyi Hospital, Beijing, 101300 China

**Keywords:** Apparent temperature, Distributed lag nonlinear model, Traumatic fracture, Emergency visits

## Abstract

**Background:**

Traumatic fractures occur frequently worldwide. However, research remains limited on the association between short-term exposure to temperature and traumatic fractures. This study aims to explore the impact of apparent temperature (AT) on emergency visits (EVs) due to traumatic fractures.

**Methods:**

Based on EVs data for traumatic fractures and the contemporary meteorological data, a generalized Poisson regression model along with a distributed lag nonlinear model (DLNM) were undertaken to determine the impact of AT on traumatic fracture EVs. Subgroup analysis by gender and age and sensitivity analysis were also performed.

**Results:**

A total of 25,094 EVs for traumatic fractures were included in the study. We observed a wide “J”-shaped relationship between AT and risk of traumatic fractures, with AT above 9.5 °C positively associated with EVs due to traumatic fractures. The heat effects became significant at cumulative lag 0–11 days, and the relative risk (RR) for moderate heat (95th percentile, 35.7 °C) and extreme heat (99.5th percentile, 38.8 °C) effect was 1.311 (95% *CI*: 1.132–1.518) and 1.418 (95% *CI*: 1.191–1.688) at cumulative lag 0–14 days, respectively. The cold effects were consistently non-significant on single or cumulative lag days across 0–14 days. The heat effects were higher among male and those aged 18–65 years old. The sensitivity analysis results remained robust.

**Conclusion:**

Higher AT is associated with cumulative and delayed higher traumatic fracture EVs. The male and those aged 18–65 years are more susceptible to higher AT.

**Supplementary Information:**

The online version contains supplementary material available at 10.1186/s12889-024-19119-z.

## Introduction

Traumatic injuries are a major cause of mortality and disability globally [1]. In China, injuries are the fifth leading cause of death in the whole population and the most common cause of death in young adults, which causes a substantial disease burden [2]. Traumatic fractures are the most common consequence of traumatic injuries and severely affect quality of life of patients. Therefore, identifying the risk factors for traumatic fractures is important to help policymakers develop prevention and control strategies.

While the individual risk factors for traumatic fractures, such as older age, occupation, smoking, excess alcohol consumption, sleeping disorder, and previous fracture history have been well elucidated [3, 4], the environmental risk factors for traumatic fractures have not been sufficiently studied. Recently, some studies have explored the association between meteorological factors, especially temperature and the incidence of hip fracture and osteoporosis fracture. The majority of these studies have consistently demonstrated that lower temperatures are associated with an increased risk of fractures [5–8]. However, the fracture types in these studies are all nontraumatic fractures, which are characterized by distinct injury mechanisms compared to traumatic fractures. Nontraumatic fractures commonly arise from low-energy injuries, whereas traumatic fractures predominantly occur as a result of high-energy injuries, such as motor vehicle collisions, falls downstairs, and sports activity [9]. Several studies have found that high temperature increased the risk of road traffic injury [10, 11]. However, there is still a lack of explicit quantification regarding the relationship between temperature and traumatic fractures.

Thus, based on emergency visits (EVs) data, our study aims to investigate the exposure-response relationship between temperature and traumatic fractures using a distributed lag nonlinear model (DLNM). Researchers have suggested that raw temperature can be misrepresentative of the true thermal effects in previous studies, because temperature perception is influenced by both actual temperature and other meteorological factors, such as humidity and wind speed [12–14]. Therefore, we employed the apparent temperature (AT) that combines ambient temperature, relative humidity, and wind speed as the indicator of heat exposure, which could better reflect the thermal sensations perceived by the human, especially in cities where the humidity is high, such as Hangzhou, China [15]. This study added to the body of knowledge on the relationship between temperature and traumatic fractures and provided a valuable reference for fracture prevention strategies.

## Methods

### Study setting

**Fig. 1:**
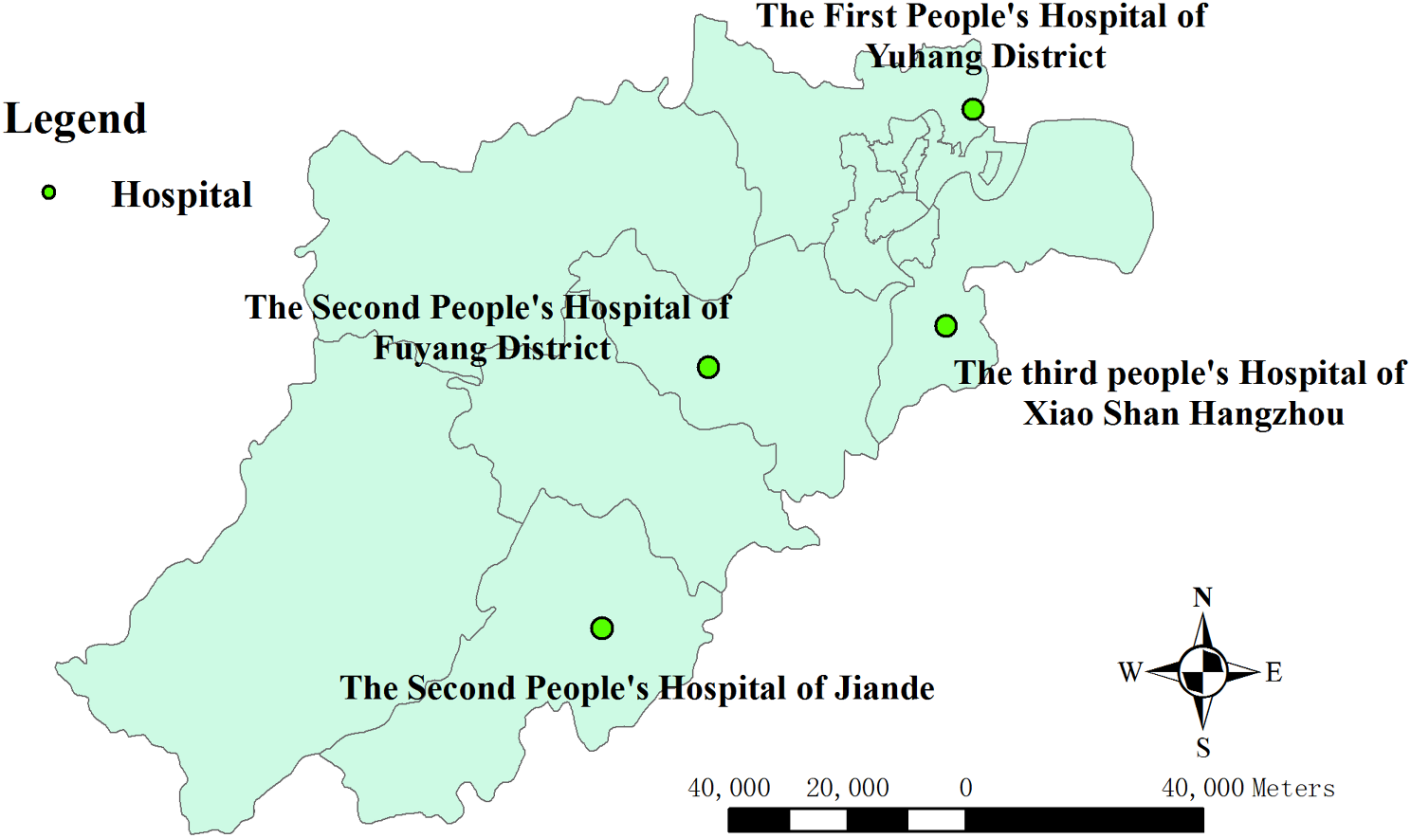
Locations of the four hospitals selected in this study in Hangzhou, China

Hangzhou, the capital of Zhejiang Province, is a typical southern city in China. There are four distinct seasons in its subtropical climate, with hot and humid summers and cold winters. The total area of the city is 16,850 km^2^ and it comprises 10 districts and three counties, with a population of 8.35 million as of 2021 [16]. The study sites selected for our study included the Third People’s Hospital of Xiaoshan District, the First People’s Hospital of Yuhang District, the Second People’s Hospital of Fuyang District, and the Second People’s Hospital of Jiande county. They are well-located in Hangzhou to serve the surrounding densely populated residential areas (Fig. 1).

### Data sources

#### Emergency visits data

Medical records were extracted from the Hospital Information System of four hospitals, covering the period from January 2017 to December 2022. The medical records included variables such as date of EVs, age, gender, residence address, fracture cause, and the principal diagnosis. Medical records that met the following criteria were included in our study: (1) The primary diagnoses were coded according to ICD-10 as follows: S02, S12, S22, S32, S42, S52, S62, S72, S82, S92, T02, T08, T10, and T12; (2) The fracture causes included road transport accidents, falls, sports injuries, being struck by an object, hit by others, and other accidental injuries. The patients who were not permanent residents of Hangzhou were excluded from this study.

#### Meteorological data

Contemporary meteorological variables including daily mean temperature, maximum temperature, minimum temperature, relative humidity, precipitation, sunshine duration, and wind speed, were obtained from China Meteorological Data Service Center (http://data.cma.cn/). AT was calculated as follows [14]:

1 $$\text{A}\text{T}=\text{T}+0.33\text{*} \text{e}-0.70\text{*}\text{W}\text{S}-4.00$$

2 $$\text{e}=\frac{\text{R}\text{H}}{100}\text{*}6.105\text{*}\text{e}\text{x}\text{p}\left(17.27\text{*}\frac{\text{T}}{237.7+\text{T}}\right)$$

where T is the daily mean temperature (°C), e is water vapor pressure (hPa), and WS is the daily mean wind speed (m/s). The e is calculated in Eq. (2) with T and RH, where RH denotes daily mean relative humidity (%).

### Statistical analysis

The demographic data on EVs for traumatic fractures and meteorological factors were summarized using a descriptive analysis. To visualize the trending of the key variables over time, a time series diagram was plotted.

Based on the evidence that the lagged and non-linear effect of temperature on nontraumatic fractures [7, 8], a DLNM with the generalized Possion Regression Model were applied to investigate the non-linear and delayed associations between AT and traumatic fracture EVs [17, 18]. To avoid to the possible multicollinearity among variables, Spearman’s correlation analysis was carried out (Supplementary Material Table S1, Figure S1), and wind speed, sunshine duration, and precipitation were identified as confounders. The model is described in Eq. (3):

3 $$\eqalign{ Log[E({Y_t})] = & \alpha + \beta *A{T_{t,l}} + ns{\rm{(}}time,df = 2*6{\rm{)}} \cr & + ns{\rm{(Pr}}{{\rm{e}}_t},df = 3{\rm{)}} + ns{\rm{(SS}}{{\rm{H}}_{\rm{t}}},df = 3{\rm{)}} \cr & + ns{\rm{(W}}{{\rm{S}}_{\rm{t}}},df = 3{\rm{)}} + \gamma *{\rm{Do}}{{\rm{w}}_{\rm{t}}} + \delta *{\rm{Holida}}{{\rm{y}}_t}\left( 3 \right) \cr}$$

where Y_t_ denotes the number of EVs for traumatic fractures on day t; α is the intercept; AT_t, l_ is the cross-basis generated from the DLNM, where l represents the number of lag days. The maximum lag day was set into 14 days. Furthermore, ns denotes the natural cubic spline function, df denotes the degrees of freedom, and time is a variable used to control for the long-term trend. According to previous literature^7^, 2 df per year for time trends was selected. Pre_t_, SSH_t_ and WS_t_ denotes precipitation, sunshine duration and wind speed on day t, and 3 df for them were used. *β, γ* and *δ* are the corresponding coefficients for AT_t, l_, the day of the week (Dow) and holidays (Holiday), respectively.

With the model mentioned above, we established the overall exposure-response relationship between AT and EV for traumatic fractures [19]. Taking the optimum AT corresponding to the lowest risk of EVs as a reference, the relative risk (RR) and 95% confidence interval (*CI*) at the 0.5th (extreme cold, -4.3 °C), 5th (moderate cold, 0.8 °C), 95th (moderate heat, 35.7 °C), and 99.5th (extreme heat, 38.8 °C) percentiles of AT were estimated, respectively. The 0.5th, 5th, 95th, and 99.5th percentiles were determined based on the distribution of daily AT data over the six-year period (2017–2022). Subgroup analyses were conducted by gender(male and female) and age group (0–17 years old, 18–65 years old, and ≥ 66 years old). All statistical analyses were performed in R software (4.2.1) with the “dlnm”, “splines”, and “ggplot2” packages. Two-sided *P*-values ≤ 0.05 were considered statistically significant.

### Sensitivity analysis

To evaluate the robustness of the main results from the model, a series of sensitivity analyses were performed, by changing the dfs for time (1–3), AT (4–6), precipitation (3–5), sunshine duration (3–5), and the maximum lag days (7 or 21 days), respectively. In addition, two different temperature indicators were compared using the cross-basis matrix of ambient temperature instead of AT. Since the sunshine duration was positively correlated with precipitation, the models excluding sunshine duration and precipitation separately were also conducted to compared with the main result.

## Results

### Descriptive statistics

Table 1 showed the basic information of daily EVs for traumatic fractures and its subgroups, as well as the daily meteorological factors between 2017 and 2022. During the study period, a total of 25,094 EVs for traumatic fractures were recorded, with an average of 11 patients visiting emergency rooms per day. Of all these cases, the proportion of EVs for the male (61.62%) was more than that for the female (38.38%). Patients aged 18–65 years accounted for 72.86%, which was higher than those aged ≥ 66 years old (20.04%), while the patients aged 0–17 years old only accounted for 7.10%. For meteorological factors, the medians of AT, mean temperature, maximum temperature, minimum temperature, relative humidity, wind speed, sunshine duration, and precipitation were 18.8 °C, 19.1 °C, 24.0 °C, 15.3 °C, 76%, 2.05 m/s, 4.2 h, and 0.1 mm, respectively.

**Table 1:** Descriptive statistics of daily EVs for patients with traumatic fractures in Hangzhou, China during 2017–2022

Variables	Total	Range	Percentage		
			*P*_25_	*P*_50_	*P*_75_
EVs	25,094	0–32	8	11	15
Gender					
Male	15,464	0–21	4	7	10
Female	9,630	0–17	3	4	6
Age					
0–17 years old	1,782	0–7	0	1	1
18–65 years old	18,283	0–27	5	8	11
≥ 66 years old	5,029	0–11	1	2	3
Meteorological factors					
AT (°C)	-	-8.3-39.7	8.6	18.8	28.7
Tmean (°C)	-	-2.7-34.7	10.8	19.1	25.8
Tmax (°C)	-	0.2–41.6	15.7	24.0	31.0
Tmin (°C)	-	-6.2-30.5	7.9	15.3	22.3
Relative humidity (%)	-	30.5–98.6	65.5	75.9	87.0
Wind speed (m/s)	-	1.1–5.9	1.8	2.1	2.4
Sunshine duration (h)	-	0.0-12.7	0.5	4.2	8.3
Precipitation (mm)	-	0.0-111.0	0.0	0.1	4.3

*Abbreviations* SD, standard deviation; P_*x*_, *x*-th percentile; AT, apparent temperature; Tmean, daily mean temperature; Tmax, daily maximum temperature; Tmin, daily minimum temperature

Supplementary Figure S2 showed the time trend of EVs for traumatic fractures, AT, mean temperature, precipitation, and sunshine duration. The meteorological variables showed a significant seasonal pattern, especially for AT and mean temperature. The daily EVs showed a discernible seasonal pattern and slightly increased with temperature. As shown in Supplementary Figure S1 and Table S1, daily mean AT was strongly and positively associated with daily mean temperature, maximum temperature, and minimum temperature, while sunshine duration was negatively correlated with precipitation and relative humidity.

### Effects of AT on EVs for traumatic fractures

#### Overall effect of AT on EVs for traumatic fractures

**Fig. 2:**
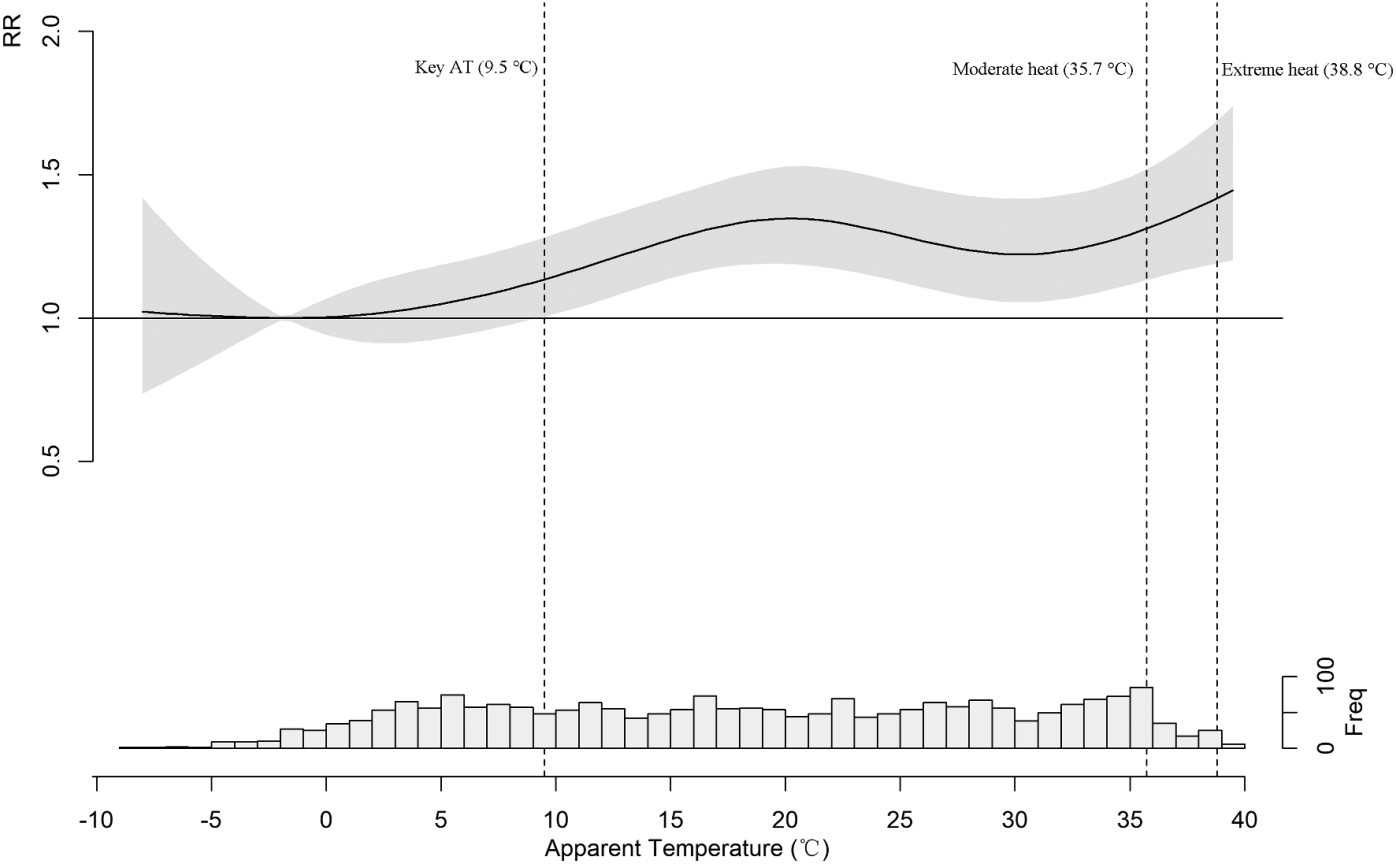
Overall exposure-response relationship between AT and EVs for patients with traumatic fractures

Figure 2 showed the overall exposure-response association between AT and EVs for traumatic fractures.

Generally, the overall exposure-response curve was wide “J”-shaped. Low AT (below − 3.0 °C) appeared to have a slight effect on EVs, but the effect was not significant. The lowest risk of EVs emerged at -3.0 °C and the risk effect became significant at 9.5 °C. As AT continued to rise above 9.5 °C, the risk gradually escalated, within the range of 18 °C to 30 °C, the risk remained relatively stable, while surpassing 30 °C led to a gradual resurgence in risk.

#### Delayed effect of specific AT on EVs for traumatic fractures

Compared with the reference of -1.8 °C, the single lag effects of the 0.5th, 5th, 95th, and 99.5th percentiles of AT were presented in Supplementary Table S2. Only a slight risk effect was found at lag 11 for extreme heat (RR = 1.026, 95% CI: 1.001–1.052). The corresponding cumulative lag effects were presented in Table 2. The cumulative effects for moderate heat and extreme heat were similar, which emerged from lag 0–11 day (RR = 1.213, 95% CI: 1.015–1.450; RR = 1.260, 95% CI: 1.027–1.546) and peaked at lag 0–14 day (RR = 1.311, 95% *CI*: 1.132–1.518; RR = 1.418, 95% *CI*: 1.191–1.688). However, no significant effects for moderate and extreme cold were identified at single and cumulative lag days.

**Table 2:** Cumulative heat and cold effects of specific ATs on EVs for traumatic fracture

Cumulative lag day(s)	RR (95%CI)			
	Extreme Cold (*P*_0.5_: -4.3℃)	Moderate Cold (*P*_5_:0.8℃)	Moderate Heat (*P*_95_: 35.78℃)	Extreme Heat (*P*_99.5_: 38.8℃)
Lag 0–0	0.989 (0.955–1.023)	1.006 (0.983–1.031)	1.046 (0.970–1.127)	1.046 (0.957–1.143)
Lag 0–1	0.978 (0.924–1.036)	1.013 (0.973–1.054)	1.081 (0.955–1.224)	1.082 (0.933–1.254)
Lag 0–2	0.970 (0.903–1.041)	1.018 (0.969–1.070)	1.108 (0.951–1.291)	1.108 (0.925–1.327)
Lag 0–3	0.962 (0.890–1.040)	1.024 (0.970–1.081)	1.126 (0.953–1.331)	1.127 (0.927–1.370)
Lag 0–4	0.956 (0.881–1.038)	1.028 (0.971–1.088)	1.139 (0.958–1.354)	1.140 (0.933–1.393)
Lag 0–5	0.952 (0.875–1.037)	1.032 (0.973–1.094)	1.148 (0.962–1.369)	1.151 (0.941–1.407)
Lag 0–6	0.950 (0.869–1.038)	1.034 (0.973–1.099)	1.154 (0.965–1.381)	1.160 (0.947–1.422)
Lag 0–7	0.950 (0.865–1.042)	1.036 (0.971–1.104)	1.161 (0.966–1.395)	1.171 (0.951–1.442)
Lag 0–8	0.951 (0.862–1.049)	1.036 (0.968–1.108)	1.169 (0.968–1.410)	1.185 (0.957–1.466)
Lag 0–9	0.954 (0.861–1.057)	1.035 (0.964–1.110)	1.179 (0.975–1.426)	1.203 (0.970–1.493)
Lag 0–10	0.960 (0.864–1.067)	1.032 (0.960–1.110)	1.194 (0.990–1.439)	1.228 (0.992–1.520)
Lag 0–11	0.968 (0.869–1.077)	1.028 (0.954–1.107)	**1.213 (1.015–1.450)**	**1.260 (1.027–1.546)**
Lag 0–12	0.978 (0.877–1.090)	1.022 (0.948–1.102)	**1.239 (1.051–1.460)**	**1.302 (1.077–1.573)**
Lag 0–13	0.990 (0.885–1.107)	1.015 (0.940–1.096)	**1.271 (1.094–1.477)**	**1.354 (1.137–1.612)**
Lag 0–14	1.005 (0.892–1.132)	1.006 (0.926–1.093)	**1.311 (1.132–1.518)**	**1.418 (1.191–1.688)**

Note Results in bold are statistically significant (*P* < 0.05)

### Subgroup analysis

The overall exposure-response relationships between AT and EVs for traumatic fractures varied in subgroups (Supplementary Figure S3–S4). Both groups of gender showed similar exposure-response curves, but the female did not show significant overall effects. In terms of age groups, the patients aged 0–17 and 18–65 years old were relatively more vulnerable to high AT.

**Fig. 3:**
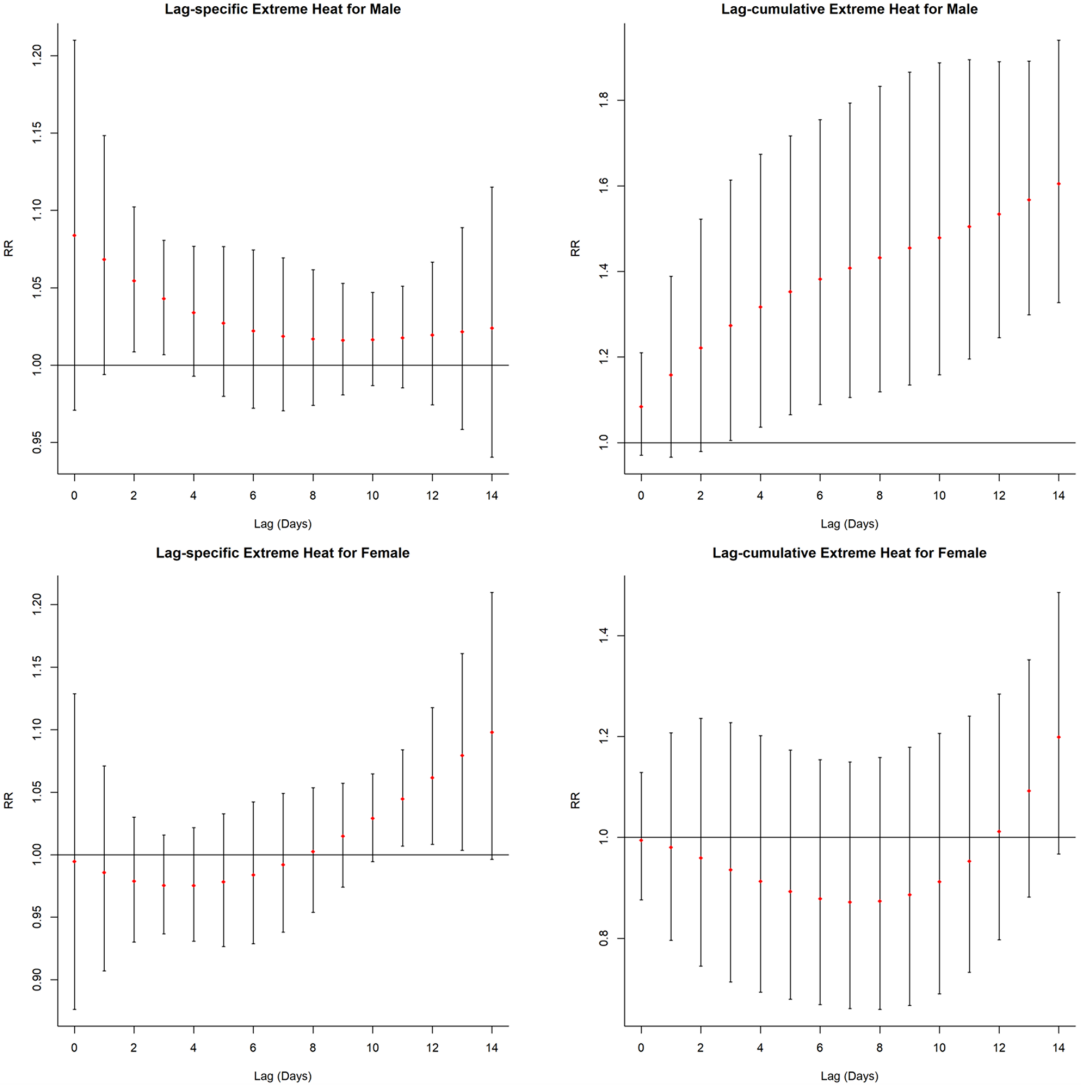
Extreme heat effects (38.8 °C vs. -1.8 °C) for daily EVs of traumatic fracture patients across different lag days in the gender groups

**Fig. 4:**
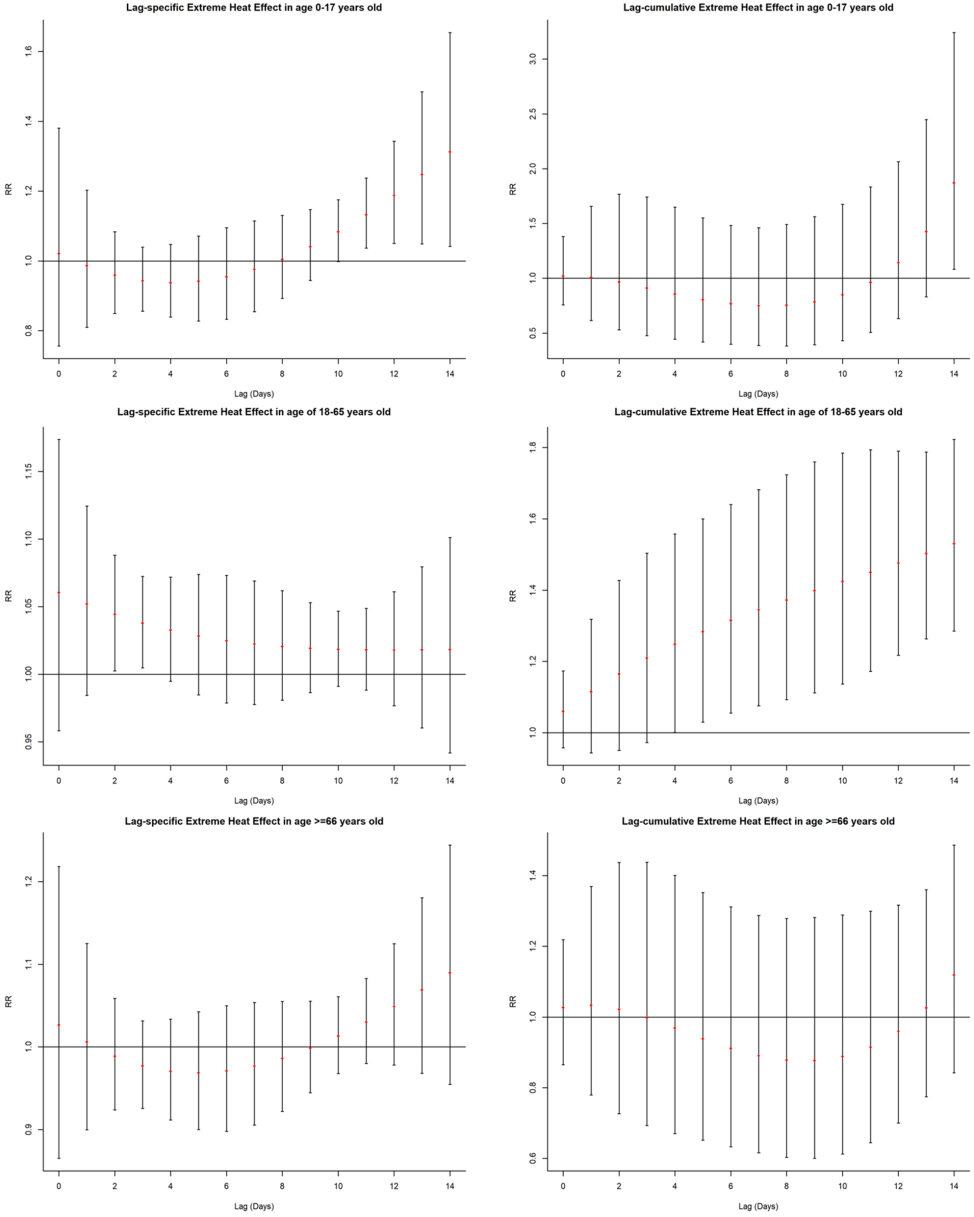
Extreme heat effects (38.8 °C vs. -1.8 °C) on daily EVs for traumatic fracture patients across different lag days in the age groups

The single and cumulative lag effects for extreme heat in subgroups were presented in Supplementary Table S3-S4, Figs. 3 and 4. In the gender subgroup, the lag-specific extreme heat effects for the male were only significant at lag 2 day (RR = 1.054, 95% *CI*: 1.009–1.102) and lag 3 day (RR = 1.043, 95% *CI*: 1.007–1.081). The lag-cumulative extreme heat effects for male became significant at lag 0–3 (RR = 1.274, 95% *CI*: 1.005–1.613) and reached the highest (RR = 1.605, 95% *CI*: 1.327–1.941) at lag 0–14 day. For the female, significant lag-specific extreme heat effects were only found at lag 11 day to lag 13 day, and the lag-cumulative extreme heat effects were non-significant.

For the patients aged 0–17 years old, the lag-specific extreme heat effects emerged at lag 11 day (RR = 1.133, 95% *CI*: 1.037–1.237) and lasted until lag 14 day (RR = 1.312, 95% *CI*: 1.041–1.654). The lag-cumulative extreme heat effect was only observed at lag 0–14 day (RR = 1.872, 95% *CI*: 1.081–3.242). For the patients aged 18–65 years old, lag-specific extreme heat effects were only found at lag 2 day (RR = 1.044, 95% *CI*: 1.002–1.088) and lag 3 day (RR = 1.038, 95% *CI*: 1.005–1.072). The lag-cumulative extreme heat effects became significant at lag 0–4 (RR = 1.249, 95% *CI*: 1.001–1.558) and peaked at lag 0–14 day (RR = 1.530, 95% *CI*: 1.285–1.822). For the patients aged ≥ 66 years old, the single and cumulative extreme heat effects were not observed.

### Sensitivity analysis

As the degree of freedom of the time trend (1–3 df/year), AT (4–6 df), sunshine duration (3–5 df), and precipitation (3–5 df) was altered, the results remained robust (Supplementary Figure S5–S8). When altering the maximum lag days (7 or 21 days), the tendency barely changed (Supplementary Figure S9). Moreover, a similar exposure-response relationship was observed for the two models employing AT and mean temperature as independent variables (Supplementary Figure S10). The exposure-response curves for the two models excluding sunshine duration and precipitation separately were similar with the overall curves (Supplementary Figure S11).

## Discussion

To the best of our knowledge, this study is the first to quantify the association between AT and traumatic fractures. Using data on EVs for traumatic fractures and meteorological factors in Hangzhou, China, a wide “J”-shaped exposure-response relationship was observed between AT and the risk of traumatic fracture, wherein higher AT were associated with an increased cumulative and delayed risk of traumatic fractures. In addition, we discovered that the male and the patients aged 18–65 years old were more susceptible to high AT.

The association between temperature and fractures have been reported in previous studies. Different from our findings, most studies found a significant association between low temperature and fractures [7, 8, 20–22]. This discrepancy may be caused by the differences in fracture type. Osteoporotic fractures, i.e., non-traumatic fractures, which is common in the elderly and caused by low energy injuries such as falls, has been paid much attention in previous related studies. When the temperature is low, snow and ice tend to make roads slippery. In such weather conditions, the elderly usually wear thicker clothes, which makes them clumsier and uncoordinated [23, 24]. Therefore, they are more likely to experience fall-related osteoporotic fractures. By contrast, our study focused on traumatic fractures, which are mainly caused by high energy injuries such as road traffic injuries. Consistent with our findings, several studies showed that road traffic injuries were significantly correlated with high temperature [10, 11, 25]. We found that the male and those aged 18–65 years old were more vulnerable to high AT. The possible reason may be that these groups are more likely to be exposed to high-risk work environments, insofar as they comprise the main labor force in society, which increases the risk of kinds of traumatic fractures.

The delayed impact of elevated AT on traumatic fractures could potentially be attributed to the cumulative influence of temperature on both physical and psychological states. Extreme temperatures have been found to elicit a sequence of non-immediate responses in vulnerable populations [26], resulting in an increased rate of hospitalization among individuals with pre-existing conditions such as dehydration, ischemic stroke, mental health disorders, and cardiovascular ailments following episodes of extreme heat [27, 28]. The presence of these underlying medical conditions will inevitably augment the susceptibility to falls and accidents, consequently elevating the likelihood of experiencing traumatic fractures. In line with our research findings, Lee et al. [29] observed a notable rise in fall-related injuries during periods of elevated temperatures in South Korea. Employing a DLNM, they further investigated the delayed effects of these injuries and determined that the risk of falling increased several days subsequent to surpassing a specific temperature threshold.

For the impact of high AT on traumatic fractures, there are some possible explanations. First, high temperature leads to individual negligence, fatigue, and a decline in cognitive function [30–32]; Secondly, alcohol consumption increases on days with higher temperatures [33, 34]; In addition, the performance of the equipment and road traffic infrastructures are inhibited by high temperature, such as lower driving performance, larger steering adjustments of the vehicle, and loss of signals [35, 36]. The above reasons all increase the probability of injuries-related fracture. It is worth noting that when the temperature is suitable, the risk of fracture also slightly increases. One possible explanation is that suitable temperatures promote people to spend more time outdoor physical activity, resulting in an increased probability of injury-related fracture [37].

There are several strengths to this study. First, to our knowledge it is the first to examine the quantitative relationship between AT and traumatic fractures. Second, the EVs as the outcome indicator could more sensitively capture the acute association between temperature and fractures than outpatient and inpatient data. Several limitations need to be acknowledged. First, the hospitals selected in our study did not include all medical institutions across Hangzhou, resulting in a limited sample representation. However, as the four study sites are evenly distributed geographically in Hangzhou, this may have reduced selection bias to some extent. Second, meteorological variables were collected based on fixed monitoring stations. The use of these data may not reflect actual personal exposure, which can lead to exposure measurement bias. Finally, other confounding factors such as individual socioeconomic status, comorbidity and fracture patterns were not taken into consideration, due to the ecological design.

## Conclusion

In conclusion, our study has found that increased AT is positively associated with traumatic fracture EVs. To mitigate the occurrence of traumatic fractures, it is imperative to implement targeted public health policies encompassing fracture prevention strategies, including but not limited to curbing outdoor activities during hot weather, developing high temperature warning systems, and optimizing medical resource allocation. The future research, encompassing multiple cities and examining various subtypes of traumatic fractures while accounting for different confounding variables, is anticipated to yield a more comprehensive understanding of the impact of AT on traumatic fracture.

### Electronic supplementary material

Below is the link to the electronic supplementary material.

Supplementary Material 1

## Data Availability

The datasets used and/or analysed during the current study available from the corresponding author on reasonable request.
