# Supplementary material for: The impact of apparent temperature on the emergency visits for traumatic fractures in Hangzhou, China
Source: BMC Public Health. 2024 Jun 24;24:1681. doi: 10.1186/s12889-024-19119-z (PMC11197263; doi:10.1186/s12889-024-19119-z)
Supplement: Supplementary file 1 — Supplementary Material 1 [file 12889_2024_19119_MOESM1_ESM.docx]

**Contents**

[**Supplementary Figure S1.** **The result of correlation analysis of meteorological factors** 2](#_Toc159707931)

[**Supplementary Table S1. The result of correlation analysis of meteorological factors** 2](#_Toc159707932)

[**Supplementary Figure S2. Time series plots for daily EVs for traumatic fractures and meteorological variables in Hangzhou, China, from 2017 to 2022.** 3](#_Toc159707933)

[**Supplementary Table S2 Heat and cold effect of specific ATs on fracture EDVs at different lag days** 4](#_Toc159707934)

[**Supplementary Table S3. The single-day lag effects of extreme heat in different groups** 5](#_Toc159707935)

[**Supplementary Table S4. The cumulative day lag effects of extreme heat in different groups** 6](#_Toc159707936)

[**Supplementary Figure S3. Overall cumulative relative risks (RRs) of AT on EDVs for fractures across lag 0–14 days (with 95% CI, shaded gray) stratified by gender** 7](#_Toc159707937)

[**Supplementary Figure S4. Overall cumulative relative risks (RRs) of AT on EDVs for fractures across lag 0–14 days (with 95% CI, shaded gray) stratified by age group** 8](#_Toc159707938)

[**Supplementary Figure S5. Sensitivity analysis when altering the degrees of freedom (df = 1–3) for controlling for the long-term trend and seasonality in the model** 9](#_Toc159707939)

[**Supplementary Figure S6. Sensitivity analysis when altering the degrees of freedom (df = 4–6) for AT in the model** 9](#_Toc159707940)

[**Supplementary Figure S7. Sensitivity analysis when altering the degrees of freedom (df = 3–5) for precipitation in the model** 10](#_Toc159707941)

[**Supplementary Figure S8. Sensitivity analysis when altering the degrees of freedom (df = 3–5) for sunshine duration in the model** 10](#_Toc159707942)

[**Supplementary Figure S9. Sensitivity analysis when changing the maximum lag day into 7 and 21 in the model** 11](#_Toc159707943)

[**Supplementary Figure S10. Comparison between models adopting AT and daily mean temperature as independent variable, respectively** 11](#_Toc159707944)

[**Supplementary Figure S11. Comparison between models without SSH and without precipation, respectively** 12](#_Toc159707945)


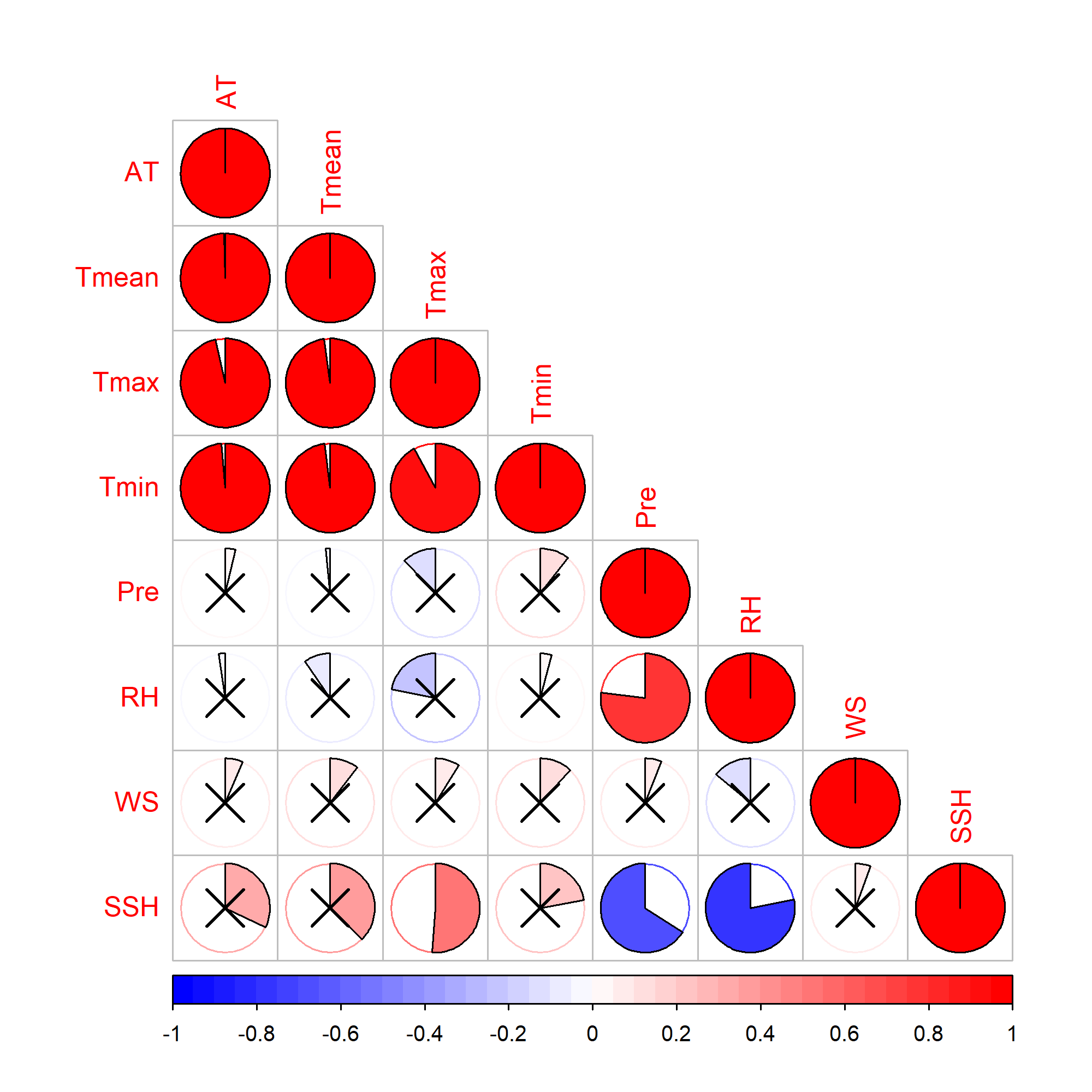


**Supplementary Figure S1.** **The result of correlation analysis of meteorological factors**

**Supplementary Table S1. The result of correlation analysis of meteorological factors**

|  | AT | Tmean | Tmax | Tmin | Pre | RH | WS | SSH |
| --- | --- | --- | --- | --- | --- | --- | --- | --- |
| AT | 1.000 | 0.996^**^ | 0.964^**^ | 0.986^**^ | 0.037 | -0.024 | 0.064 | 0.323 |
| Tmean | 0.996^**^ | 1.000 | 0.977^**^ | 0.980^**^ | -0.016 | -0.096 | 0.103 | 0.374 |
| Tmax | 0.964^**^ | 0.977^**^ | 1.000 | 0.921^**^ | -0.121 | -0.220 | 0.090 | 0.511^*^ |
| Tmin | 0.986^**^ | 0.980^**^ | 0.921^**^ | 1.000 | 0.106 | 0.040 | 0.118 | 0.221 |
| Pre | 0.037 | -0.016 | -0.121 | 0.106 | 1.000 | 0.769^**^ | 0.059 | -0.659^**^ |
| RH | -0.024 | -0.096 | -0.220 | 0.040 | 0.769^**^ | 1.000 | -0.139 | -0.780^**^ |
| WS | 0.064 | 0.103 | 0.090 | 0.118 | 0.059 | -0.139 | 1.000 | 0.056 |
| SSH | 0.323 | 0.374 | 0.511^*^ | 0.221 | -0.659^**^ | -0.780^**^ | 0.056 | 1.000 |

**P value＜0.01，* P value＜0.05；AT, apparent temperature; Tmean, daily mean temperature; Tmax, daily maximum temperature; Tmin, daily minimum temperature; RH, Relative humidity; WS: Wind speed; SD: sunshine duration

**
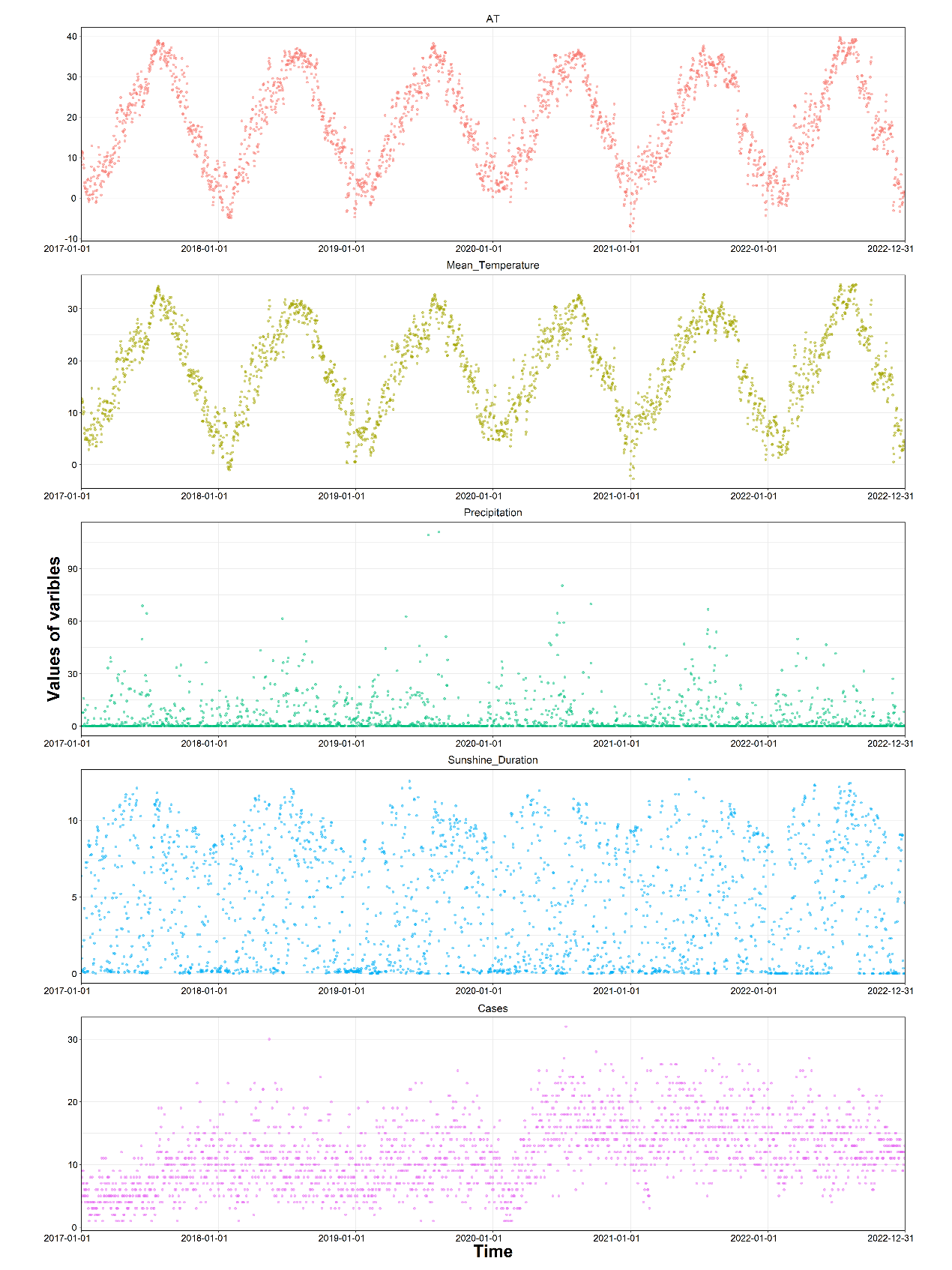
**

**Supplementary Figure S2. Time series plots for daily EVs for traumatic fractures and meteorological variables in Hangzhou, China, from 2017 to 2022.**

**Supplementary Table S2 Heat and cold effect of specific ATs on fracture EDVs at different lag days**

| lag day | RR (*95%CI*) | | | |
| --- | --- | --- | --- | --- |
|  | Extreme Cold | Moderate Cold | Moderate Heat | Extreme Heat |
|  | (P_0.5_: -4.34℃) | (P_5_:0.79℃) | (P_95_: 35.70℃) | (P_99.5_: 38.79℃) |
| lag 0 | 0.989 (0.955-1.023) | 1.006 (0.983-1.031) | 1.046 (0.970-1.127) | 1.046 (0.957-1.143) |
| lag 1 | 0.990 (0.967-1.013) | 1.006 (0.990-1.023) | 1.034 (0.984-1.088) | 1.034 (0.975-1.097) |
| lag 2 | 0.991 (0.976-1.006) | 1.006 (0.995-1.017) | 1.024 (0.992-1.058) | 1.024 (0.987-1.063) |
| lag 3 | 0.992 (0.980-1.005) | 1.005 (0.996-1.014) | 1.017 (0.990-1.044) | 1.017 (0.987-1.048) |
| lag 4 | 0.994 (0.980-1.008) | 1.004 (0.995-1.014) | 1.011 (0.983-1.040) | 1.012 (0.979-1.046) |
| lag 5 | 0.996 (0.980-1.011) | 1.003 (0.993-1.014) | 1.008 (0.976-1.040) | 1.009 (0.971-1.048) |
| lag 6 | 0.998 (0.981-1.014) | 1.002 (0.991-1.014) | 1.006 (0.973-1.040) | 1.008 (0.969-1.050) |
| lag 7 | 0.999 (0.984-1.015) | 1.001 (0.990-1.012) | 1.006 (0.974-1.039) | 1.009 (0.971-1.049) |
| lag 8 | 1.001 (0.987-1.016) | 1.000 (0.990-1.010) | 1.007 (0.978-1.036) | 1.012 (0.978-1.047) |
| lag 9 | 1.004 (0.991-1.016) | 0.999 (0.990-1.007) | 1.009 (0.986-1.033) | 1.016 (0.987-1.045) |
| lag 10 | 1.006 (0.995-1.017) | 0.997 (0.990-1.005) | 1.012 (0.993-1.032) | 1.021 (0.997-1.044) |
| lag 11 | 1.008 (0.996-1.021) | 0.996 (0.987-1.005) | 1.016 (0.995-1.038) | **1.026 (1.001-1.052)** |
| lag 12 | 1.010 (0.994-1.027) | 0.994 (0.983-1.006) | 1.021 (0.991-1.052) | 1.033 (0.997-1.070) |
| lag 13 | 1.013 (0.991-1.035) | 0.993 (0.978-1.008) | 1.026 (0.984-1.070) | 1.040 (0.989-1.093) |
| lag 14 | 1.015 (0.987-1.044) | 0.991 (0.972-1.011) | 1.031 (0.975-1.090) | 1.047 (0.980-1.120) |

**Supplementary Table S3. The single-day lag effects of extreme heat in different groups**

| Lag day | Gender | |  | Age | | |
| --- | --- | --- | --- | --- | --- | --- |
|  | Male | Female |  | 0-17 | 18-65 | ≥66 |
| Lag0 | 1.084 (0.971-1.210) | 0.994 (0.876-1.129) |  | 1.022 (0.756-1.380) | 1.060 (0.958-1.174) | 1.027 (0.865-1.218) |
| Lag1 | 1.068 (0.994-1.148) | 0.986 (0.907-1.071) |  | 0.987 (0.810-1.202) | 1.052 (0.984-1.125) | 1.006 (0.900-1.125) |
| Lag2 | **1.054 (1.009-1.102)** | 0.979 (0.930-1.030) |  | 0.959 (0.849-1.084) | **1.044 (1.002-1.088)** | 0.989 (0.924-1.059) |
| Lag3 | **1.043 (1.007-1.081)** | 0.975 (0.937-1.016) |  | 0.943 (0.856-1.039) | **1.038 (1.005-1.072)** | 0.977 (0.926-1.032) |
| Lag4 | 1.034 (0.993-1.077) | 0.975 (0.931-1.022) |  | 0.938 (0.839-1.048) | 1.033 (0.995-1.072) | 0.971 (0.912-1.034) |
| Lag5 | 1.027 (0.980-1.077) | 0.978 (0.927-1.033) |  | 0.942 (0.828-1.071) | 1.028 (0.985-1.074) | 0.969 (0.900-1.043) |
| Lag6 | 1.022 (0.972-1.075) | 0.984 (0.929-1.042) |  | 0.955 (0.833-1.095) | 1.025 (0.979-1.073) | 0.971 (0.898-1.050) |
| Lag7 | 1.019 (0.970-1.069) | 0.992 (0.938-1.049) |  | 0.976 (0.855-1.114) | 1.022 (0.978-1.069) | 0.977 (0.906-1.054) |
| Lag8 | 1.017 (0.974-1.062) | 1.002 (0.954-1.053) |  | 1.005 (0.893-1.130) | 1.020 (0.981-1.062) | 0.986 (0.922-1.055) |
| Lag9 | 1.016 (0.981-1.053) | 1.015 (0.974-1.057) |  | 1.041 (0.944-1.147) | 1.019 (0.986-1.053) | 0.998 (0.945-1.055) |
| Lag10 | 1.016 (0.987-1.047) | 1.029 (0.995-1.065) |  | 1.083 (0.999-1.175) | 1.018 (0.991-1.047) | 1.013 (0.968-1.061) |
| Lag11 | 1.018 (0.985-1.051) | **1.045 (1.007-1.084)** |  | **1.133 (1.037-1.237)** | 1.018 (0.988-1.049) | 1.030 (0.980-1.083) |
| Lag12 | 1.019 (0.974-1.067) | **1.062 (1.008-1.118)** |  | **1.188 (1.050-1.343)** | 1.018 (0.977-1.061) | 1.049 (0.978-1.125) |
| Lag13 | 1.022 (0.958-1.089) | **1.079 (1.004-1.161)** |  | **1.248 (1.049-1.484)** | 1.018 (0.960-1.080) | 1.069 (0.968-1.180) |
| Lag14 | 1.024 (0.940-1.115) | 1.098 (0.996-1.210) |  | **1.312 (1.041-1.654)** | 1.018 (0.942-1.101) | 1.090 (0.955-1.244) |

**Supplementary Table S4. The cumulative day lag effects of extreme heat in different groups**

| Cumulative lag day(s) | Gender | |  | Age | | |
| --- | --- | --- | --- | --- | --- | --- |
|  | Male | Female |  | 0-17 | 18-65 | ≥66 |
| Lag 0-0 | 1.084 (0.971-1.210) | 0.994 (0.876-1.129) |  | 1.022 (0.756-1.380) | 1.060 (0.958-1.174) | 1.027 (0.865-1.218) |
| Lag 0-1 | 1.158 (0.966-1.388) | 0.980 (0.796-1.208) |  | 1.008 (0.614-1.656) | 1.116 (0.944-1.319) | 1.033 (0.779-1.369) |
| Lag 0-2 | 1.221 (0.980-1.522) | 0.959 (0.745-1.236) |  | 0.967 (0.529-1.767) | 1.165 (0.951-1.428) | 1.022 (0.726-1.437) |
| Lag 0-3 | **1.274 (1.005-1.613)** | 0.936 (0.713-1.228) |  | 0.912 (0.477-1.743) | 1.209 (0.972-1.504) | 0.998 (0.693-1.438) |
| Lag 0-4 | **1.317 (1.036-1.673)** | 0.913 (0.693-1.201) |  | 0.855 (0.443-1.650) | **1.249 (1.001-1.558)** | 0.969 (0.670-1.400) |
| Lag 0-5 | **1.352 (1.065-1.717)** | 0.893 (0.679-1.173) |  | 0.805 (0.418-1.551) | **1.284 (1.030-1.600)** | 0.939 (0.652-1.352) |
| Lag 0-6 | **1.382 (1.089-1.755)** | 0.878 (0.669-1.154) |  | 0.769 (0.399-1.483) | **1.316 (1.056-1.640)** | 0.911 (0.633-1.311) |
| Lag 0-7 | **1.408 (1.106-1.793)** | 0.871 (0.661-1.149) |  | 0.750 (0.385-1.462) | **1.345 (1.076-1.682)** | 0.890 (0.616-1.287) |
| Lag 0-8 | **1.432 (1.119-1.832)** | 0.874 (0.659-1.158) |  | 0.754 (0.381-1.490) | **1.372 (1.093-1.724)** | 0.878 (0.603-1.279) |
| Lag 0-9 | **1.455 (1.134-1.866)** | 0.887 (0.667-1.178) |  | 0.784 (0.394-1.562) | **1.399 (1.112-1.760)** | 0.877 (0.600-1.281) |
| Lag 0-10 | **1.479 (1.159-1.887)** | 0.912 (0.690-1.206) |  | 0.850 (0.431-1.675) | **1.424 (1.137-1.785)** | 0.888 (0.612-1.289) |
| Lag 0-11 | **1.505 (1.195-1.895)** | 0.953 (0.732-1.240) |  | 0.963 (0.505-1.834) | **1.450 (1.172-1.794)** | 0.915 (0.645-1.299) |
| Lag 0-12 | **1.534 (1.245-1.890)** | 1.012 (0.797-1.284) |  | 1.143 (0.633-2.064) | **1.476 (1.217-1.790)** | 0.960 (0.700-1.316) |
| Lag 0-13 | **1.567 (1.299-1.891)** | 1.092 (0.882-1.352) |  | 1.426 (0.831-2.449) | **1.503 (1.264-1.787)** | 1.026 (0.774-1.360) |
| Lag 0-14 | **1.605 (1.327-1.941)** | 1.199 (0.967-1.486) |  | **1.872 (1.081-3.242)** | **1.530 (1.285-1.822)** | 1.119 (0.842-1.486) |


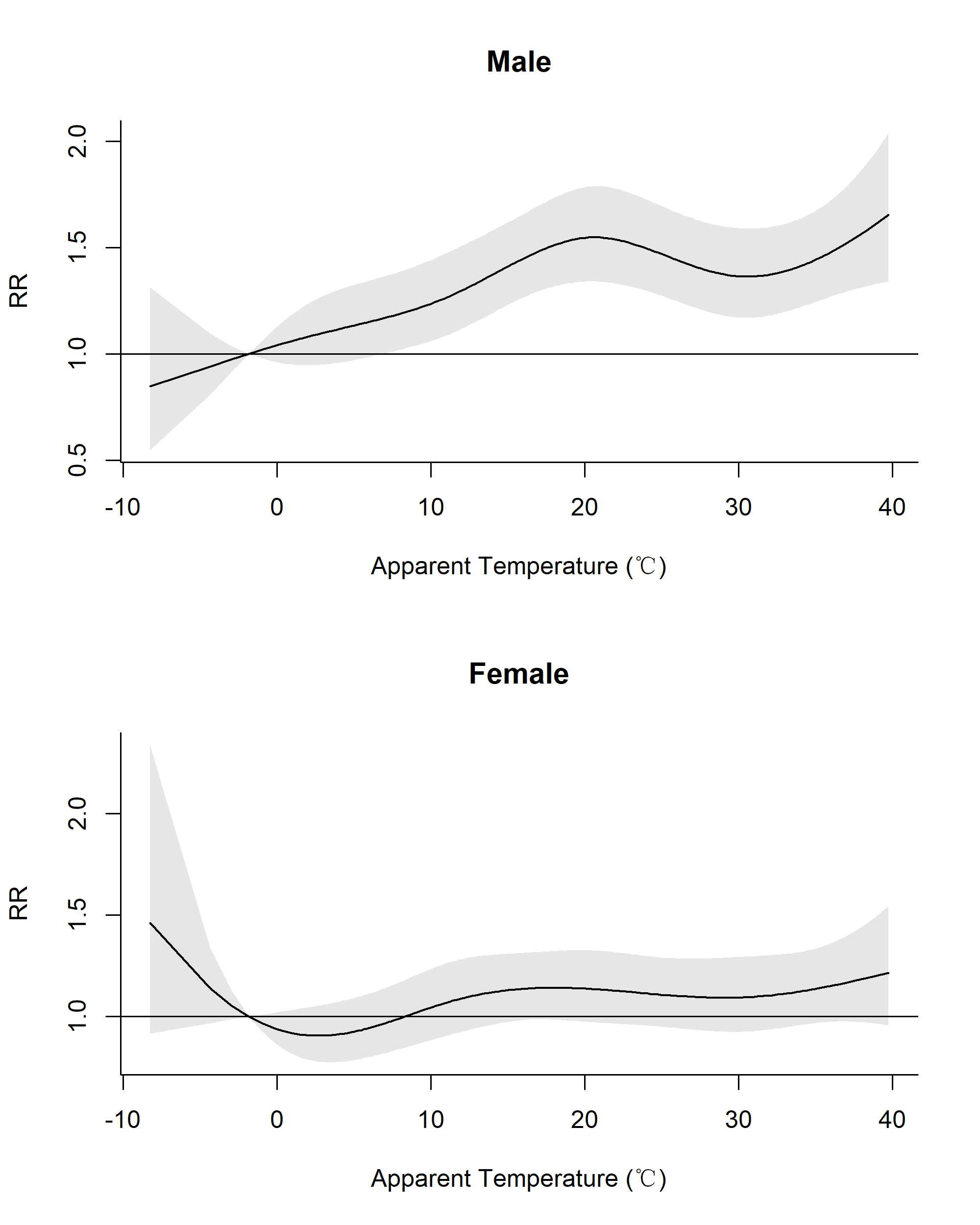


**Supplementary Figure S3. Overall cumulative relative risks (RRs) of AT on EDVs for fractures across lag 0–14 days (with 95% CI, shaded gray) stratified by gender**


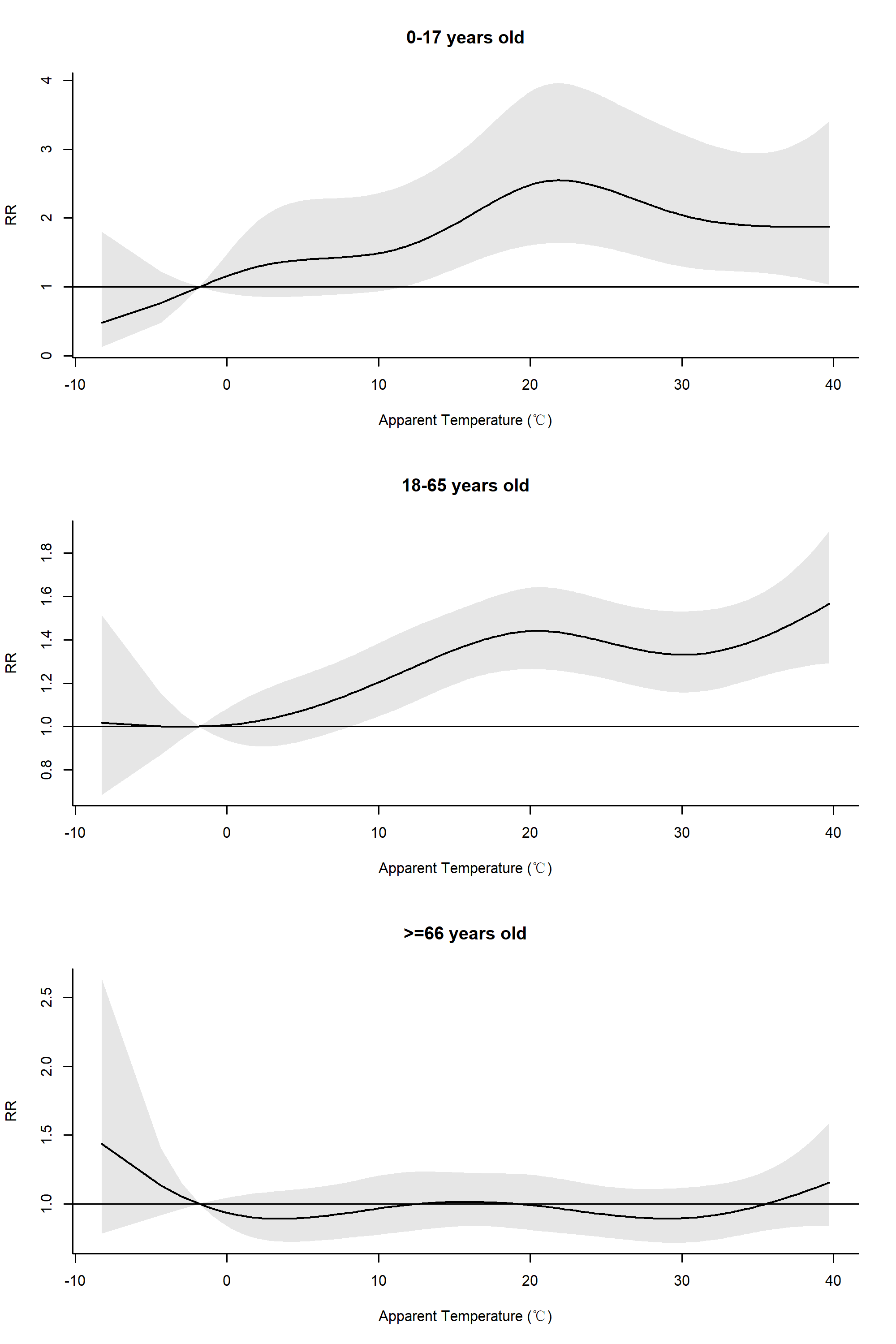


**Supplementary Figure S4. Overall cumulative relative risks (RRs) of AT on EDVs for fractures across lag 0–14 days (with 95% CI, shaded gray) stratified by age group**

**
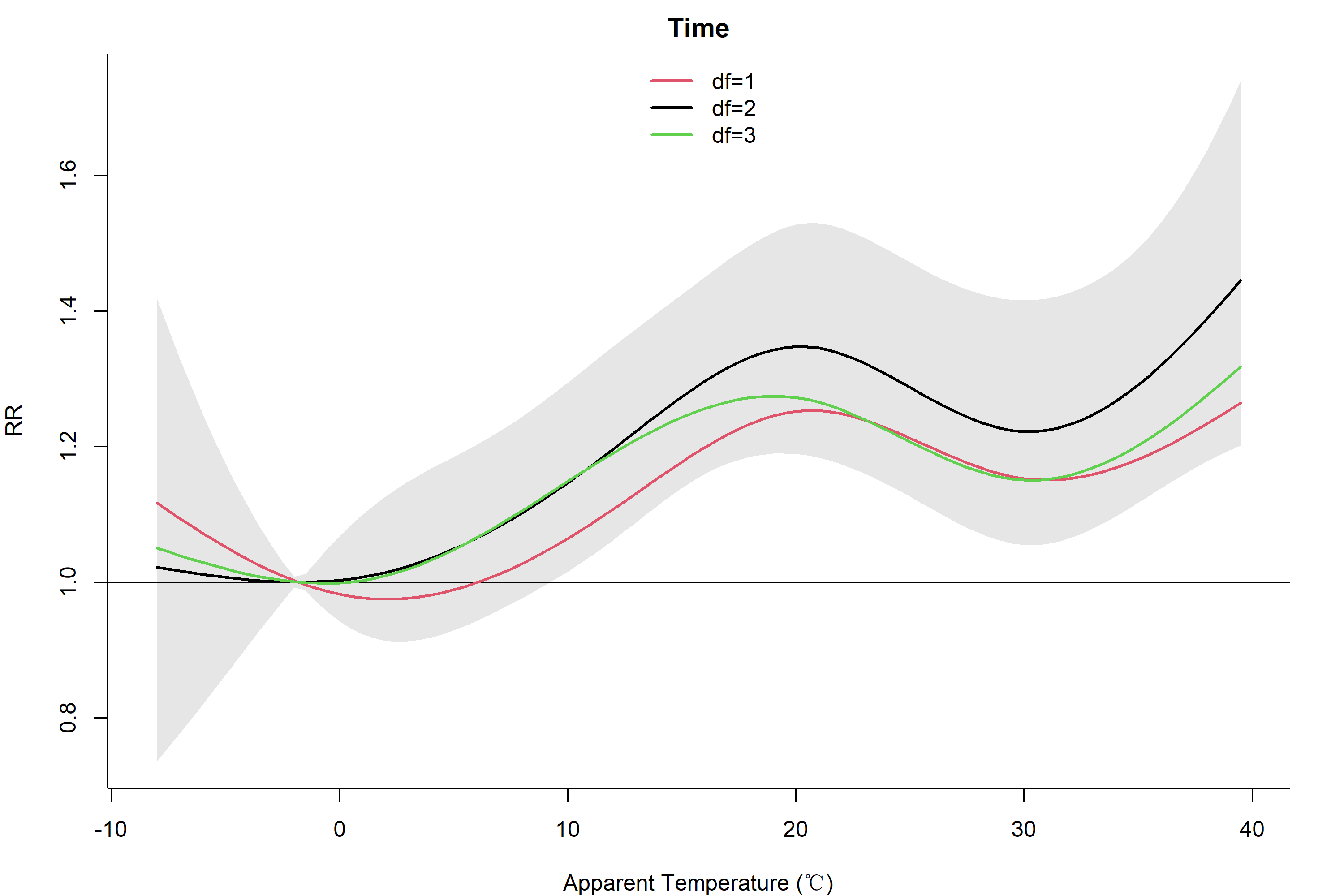
**

**Supplementary Figure S5. Sensitivity analysis when altering the degrees of freedom (df = 1–3) for controlling for the long-term trend and seasonality in the model**


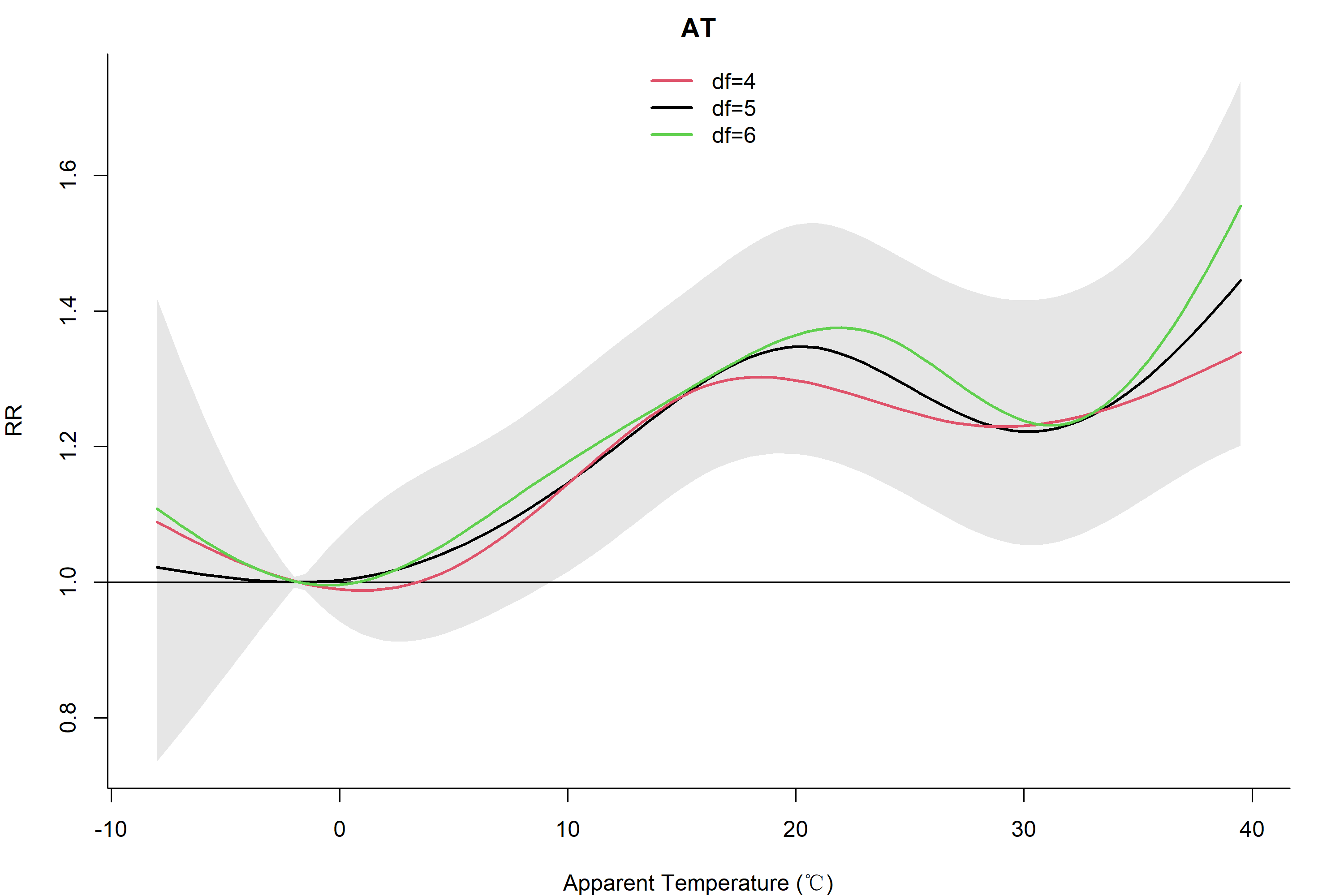


**Supplementary Figure S6. Sensitivity analysis when altering the degrees of freedom (df = 4–6) for AT in the model**


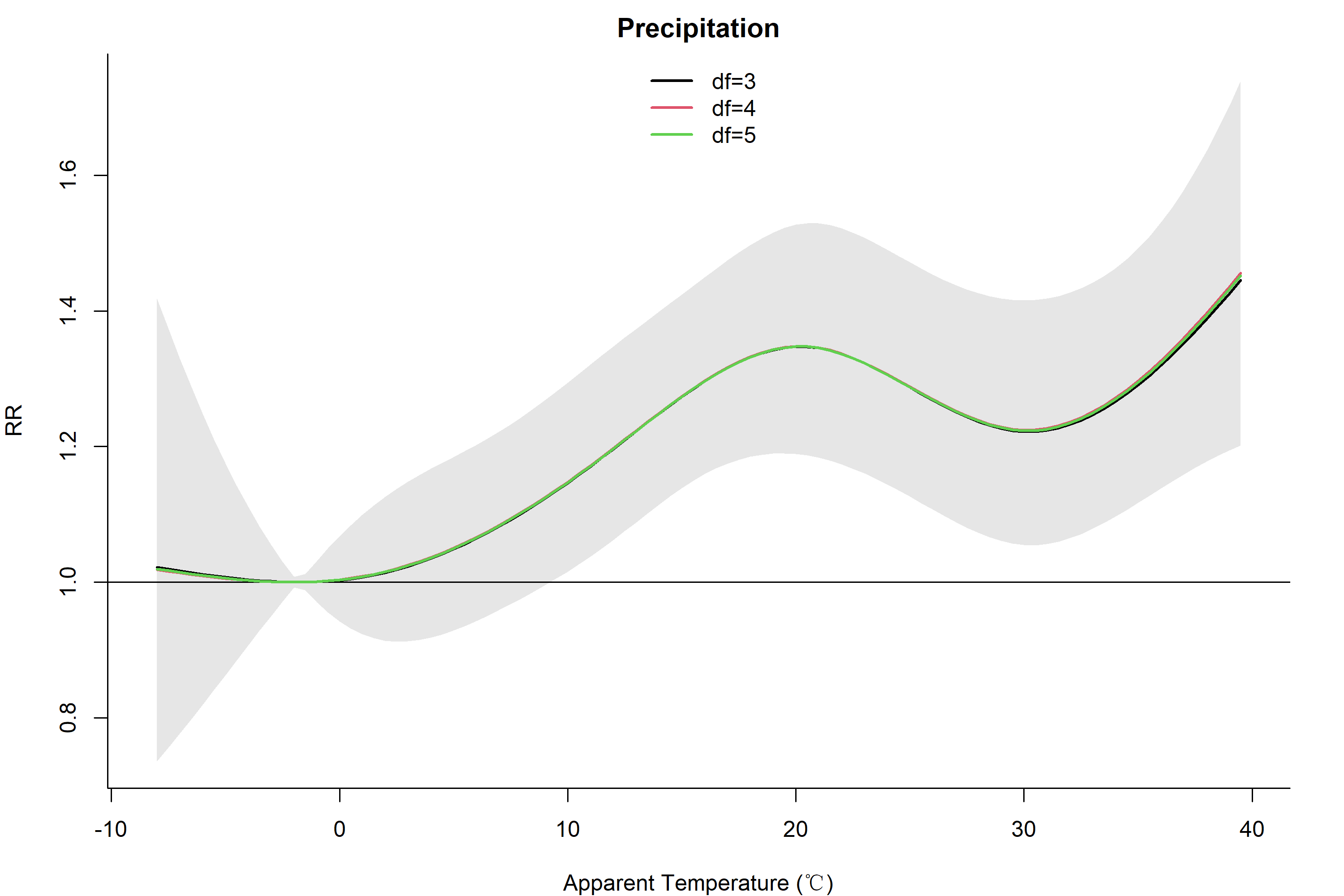


**Supplementary Figure S7. Sensitivity analysis when altering the degrees of freedom (df = 3–5) for precipitation in the model**


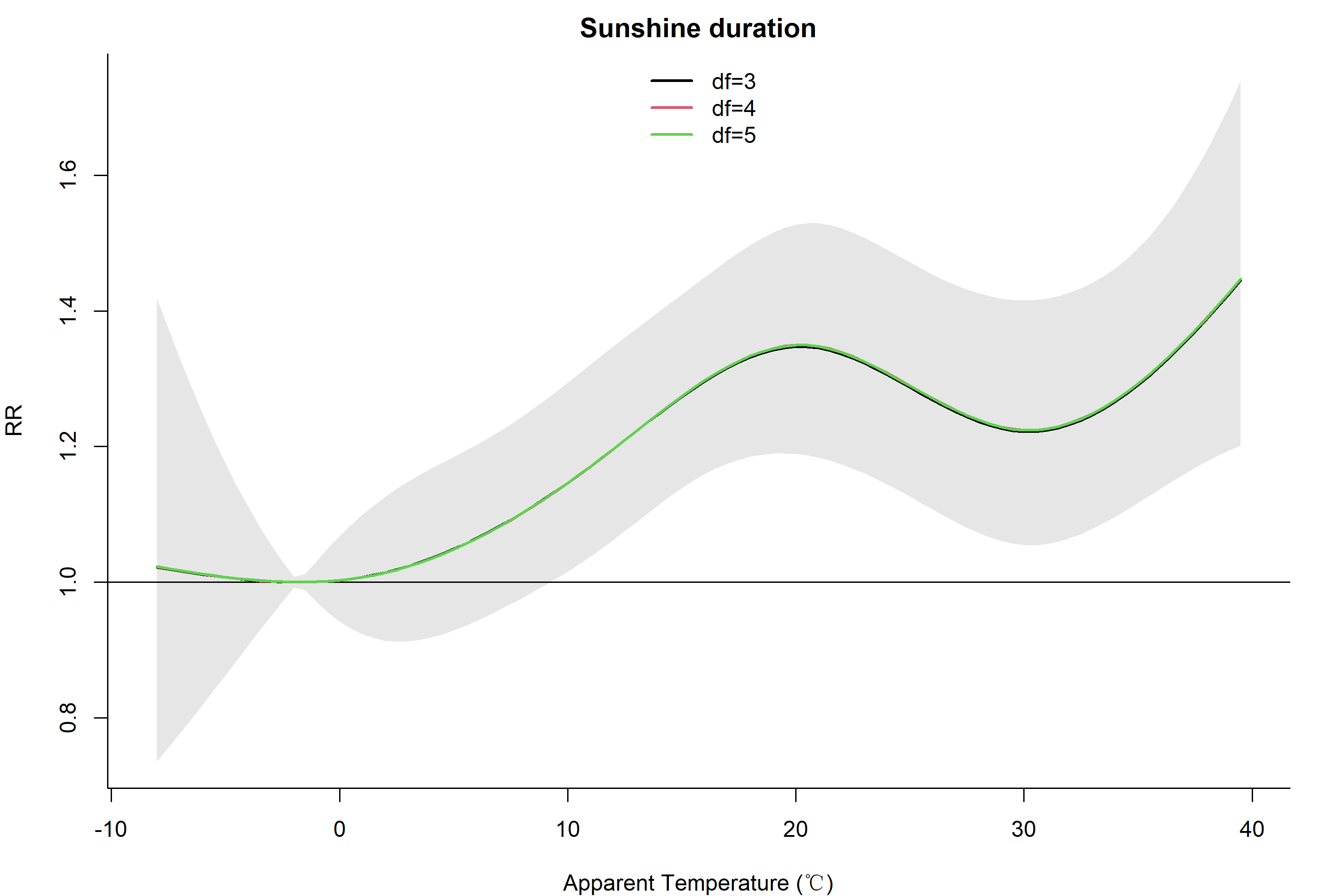


**Supplementary Figure S8. Sensitivity analysis when altering the degrees of freedom (df = 3–5) for sunshine duration in the model**


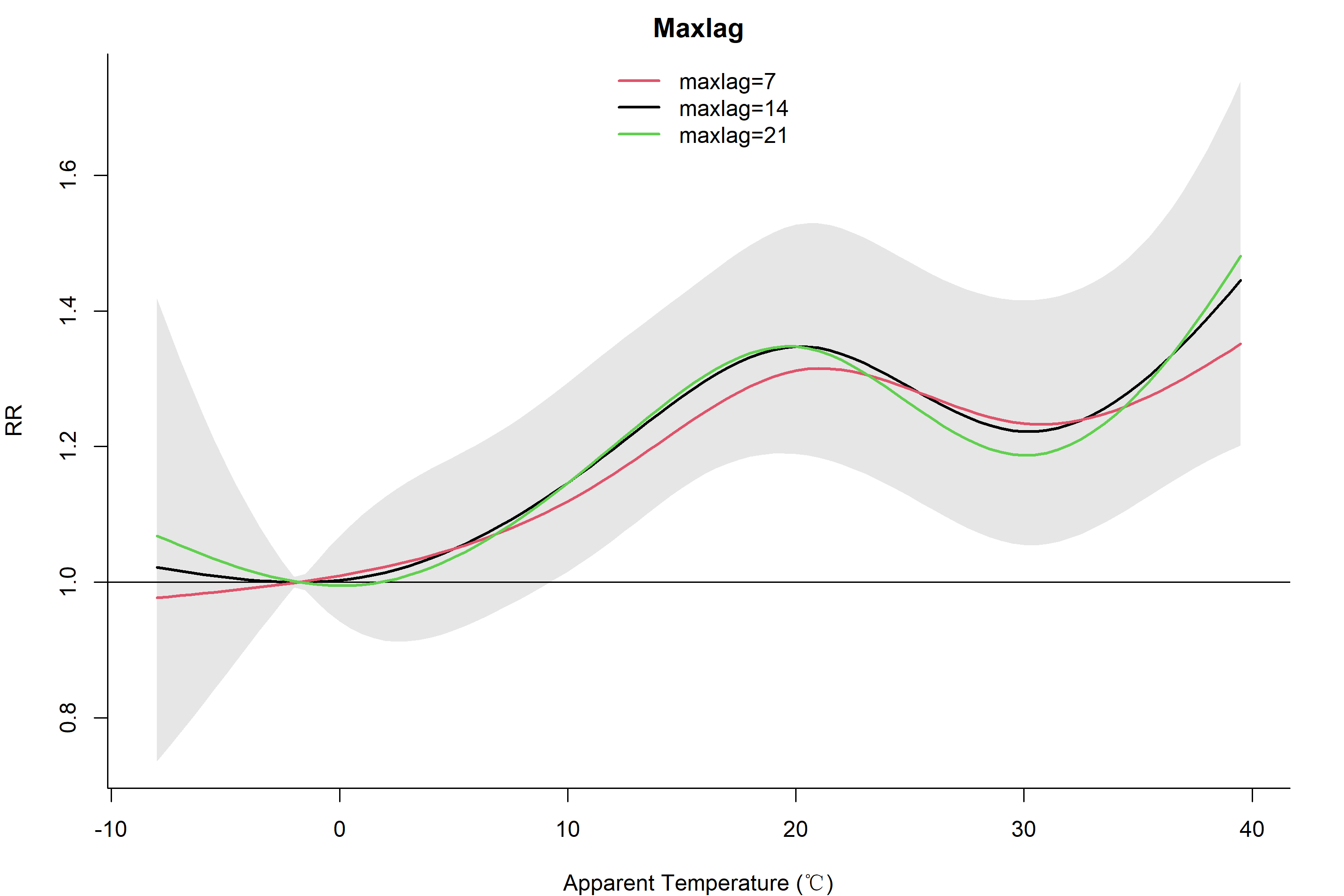


**Supplementary Figure S9. Sensitivity analysis when changing the maximum lag day into 7 and 21 in the model**


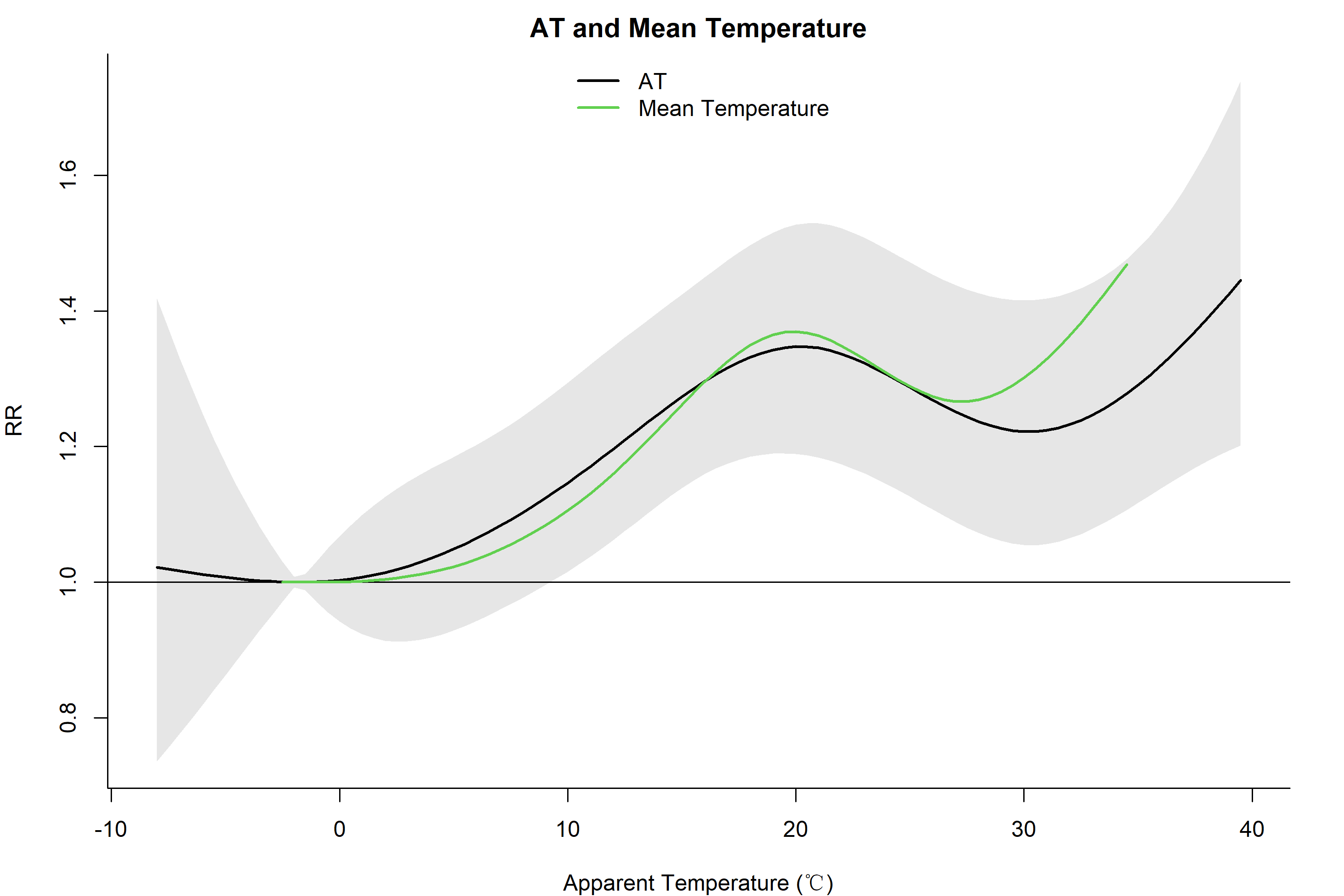


**Supplementary Figure S10. Comparison between models adopting AT and daily mean temperature as independent variable, respectively**

**
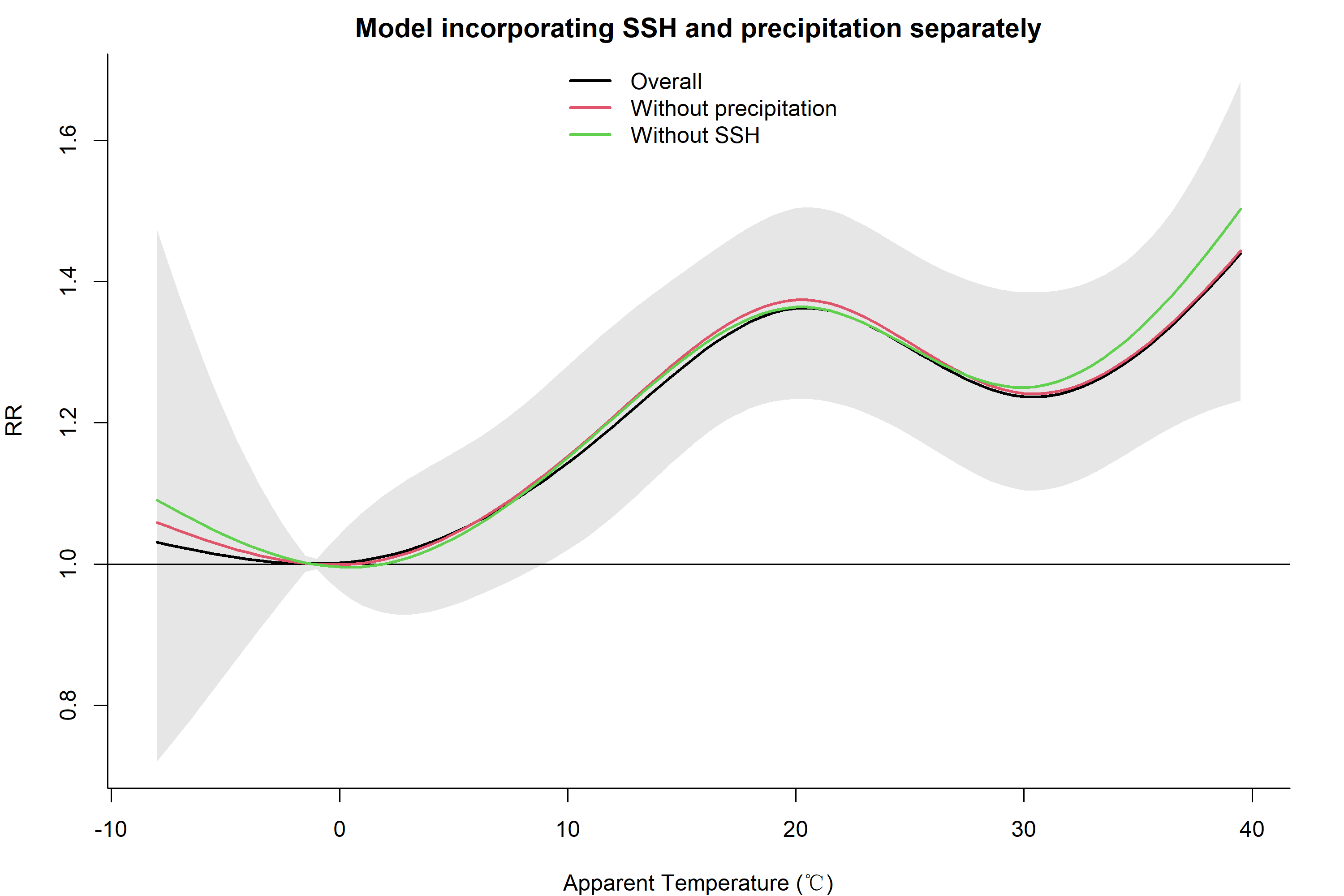
**

**Supplementary Figure S11. Comparison between models without SSH and without precipitation, respectively**
